# Patterns of sensitization to inhalant and food allergens among pediatric patients from the Moscow region (Russian Federation)

**DOI:** 10.1371/journal.pone.0194775

**Published:** 2018-03-22

**Authors:** Sergei Voloshin, Olga Smoldovskaya, Guzel Feyzkhanova, Alla Arefieva, Lyudmila Pavlushkina, Tatiana Filatova, Veronika Butvilovskaya, Marina Filippova, Yuri Lysov, Sergey Shcherbo, Alexander Makarov, Alla Rubina, Alexander Zasedatelev

**Affiliations:** 1 Engelhardt Institute of Molecular Biology, Russian Academy of Sciences, Moscow, Russia; 2 Filatov Moscow City Pediatric Clinic No. 13, Moscow, Russia; Mie Daigaku, JAPAN

## Abstract

The immunological profiles of human specific IgE (sIgE) and specific IgG4 (sIgG4) vary by genetic predisposition, living conditions in different geographical locations and patient’s age. The aim of our study was to analyze sIgE and sIgG4 patterns and their age-dependent changes in patients from the Moscow region. For identifying sIgE and sIgG4 profiles the blood samples from 513 patients aged 6 months to 17 years who were showing symptoms of allergic diseases were analyzed using microarrays containing 31 allergens. The highest sIgE prevalence was observed for birch pollen (32%) among pollen allergens, cat dander (24%) among indoor allergens, and egg whites (21%) among food allergens. The most common sIgG4 response was developed toward egg whites (80% of patients). Age-related elevation was identified for patients with increased sIgE to pollen allergens and indoor allergens (cat or dog dander and house dust mites). For each allergen, the proportion of cases with significant levels of sIgG4 appeared to increase with patients’ age. The data on allergen-specific sIgE and sIgG4 prevalence show both general trends and some local special aspects that are indicative for the Moscow region. This information should be useful in terms of epidemiology of allergic diseases.

## Introduction

Currently, up to 20% of the adult population of developed countries and up to 40–50% of school children are affected by different allergic diseases [1]. It is well known that the formation of allergic diseases is associated with the increase in skin and mucus barrier permeability and based on genetic predisposition and adverse environmental conditions [2]. Thus, the recognition of allergens leading to certain adverse symptoms in different geographical regions is becoming more important not only for general allergology but also for the development of efficient methods of allergy diagnostics.

Allergen-specific immunoglobulin E (sIgE) is the main marker of type I hypersensitivity reaction to a certain agent. sIgE bound to an allergen is involved in the process of cross-linking of FcƐRI receptors leading to basophil and mast cell activation followed by the inflammatory mediator release [2]. However, the occurrence of sIgE is not always accompanied by clinical symptoms.

Allergen-specific IgG and specifically IgG4 acting as blocking antibodies interfere IgE binding with allergens and prevent cross-linking of FcƐRI receptors and further cellular activation [3]. Unlike the other IgG subclasses, IgG4 antibodies are involved in Fab-arm exchange resulting in the occurrence of antibodies with bivalent reactivity. Bispecific antibodies unable aggregate in large complexes with allergens to activate the complement system and to induce hypersensitivity reactions of types II and III [4]. That is why IgG4 presence likely leads to reduce clinical symptoms of allergy and in some cases, can indicate a tolerance to allergens [5,6]. In combination with sIgE concentrations, the sIgG4 levels may provide more inclusive information for *in vitro* analysis interpretation [7] and is mostly used to evaluate the efficiency of allergen-specific immunotherapy [8].

Currently, there are a number of studies describing the immunological profiles of sIgE [9,10] and sIgG4 [11], especially in relation to recombinant allergens, among patients with different pathologies in various geographical locations. However, Central Russia has been investigated less in the aforementioned studies.

To fill this gap in the research, we performed a screening analysis of blood samples from a representative (513 patients) group of patients from the Moscow region, as well as from a typical urban region of Central Russia that has an adverse ecological situation. The profiles of sIgE and sIgG4 were identified with microarrays containing 31 allergens for patients aged 6 months to 17 years with allergic symptoms.

## Materials and methods

### Participants

Patients from the Filatov Moscow City Pediatric Clinic No. 13 aged 6 months to 17 completed years with suspected allergic diseases or proven allergic diagnosis (pollinosis, allergic rhinitis, atopic dermatitis and bronchial asthma) were included in the study. The continuous recruitment was carried out from May, 2016 to December, 2016. Exclusion criteria were acute infectious diseases within 2 weeks before the blood sampling and the treatment by allergen-specific immunotherapy.

After the exclusion, totally 513 patients were enrolled. The written informed consent was obtained from one of the parents or guardians of the patients. The study was approved by local ethics committee of the Filatov Moscow City Pediatric Clinic No. 13.

All the patients were divided into 6 age groups: 0–1, 2–3, 4–5, 6–8, 9–13, 14–17, each containing from 77 to 94 participants.

### Samples

The surplus of blood serum samples of participants that were left over after routine diagnostic procedures were used for the analysis of allergen-specific IgE and IgG4. The blood samples were measured once for each subject. All of the samples were exposed to a single refrigeration cycle at -45°C.

### Analysis of blood serum on microarrays

The evaluation of sIgE and sIgG4 levels was performed using multiplex fluorescent microarray assays. Microarrays were developed as the matrix of hemispherical gel pads of 0.1 nl volume [12,13], and each pad contained one of the 31 immobilized allergens (GREER, Lenoir, NC, USA).

The protocol for the analysis and verification of this method were described in previous studies [14,15,16]. Briefly the procedure of the analysis on a microarray consisted of two steps: incubation with 65 μl of serum for 20 hours at 37°C and incubation with 50 μl of developing antibodies (solution of anti-IgE-Cy5 and/or anti-IgG4-Cy3 antibodies) for 1 hour at 37°C. Between these steps microarrays were washed with PBST (0.01 M phosphate buffer, pH 7.2, 0.15 M NaCl, 0.1% Tween-20 (Sigma, USA)) for 20 min and 30 min respectively.

The registration of fluorescence signals was performed using a two-wavelength microarray analyser (EIMB RAS) based on the concept of digital wide-field fluorescence microscopy [17]. The measuring range of the method is 0.25-100 IU/ml for sIgE and 100-2500 ng/ml for sIgG4.

The patient was considered sensitized (demonstrated increased sIgE level) to the allergen if the level of sIgE to this allergen exceeded the minimal threshold of 0.35 IU/ml. The level of sIgG4 in relation to the allergen was considered to be significant if the concentration of sIgG4 was more than 110 ng/ml.

### Data processing

The prevalence of sensitized individuals and the prevalence of subjects with a significant sIgG4 level were calculated for each of the age groups as the proportion of the patients with increased sIgE and significant sIgG4 for all the patients included in the group. 95% Confidence Intervals ("exact" Clopper-Pearson confidence interval [18]) for the observed proportions were calculated by MedCalc version 14.8.1. Statistical significance of the differences in proportions between various age groups was estimated according the results of the Fisher’s exact test calculated by MedCalc. The differences were considered statistically significant for the pair-wise comparisons with p<0.05.

Diagram plotting was performed using Microsoft Excel 2010.

## Results

In this study, 513 blood serum samples were analyzed from patients aged 6 months to 17 years who were showing symptoms of allergic diseases, such as pollinosis, atopic dermatitis, and bronchial asthma.

**Fig 1:**
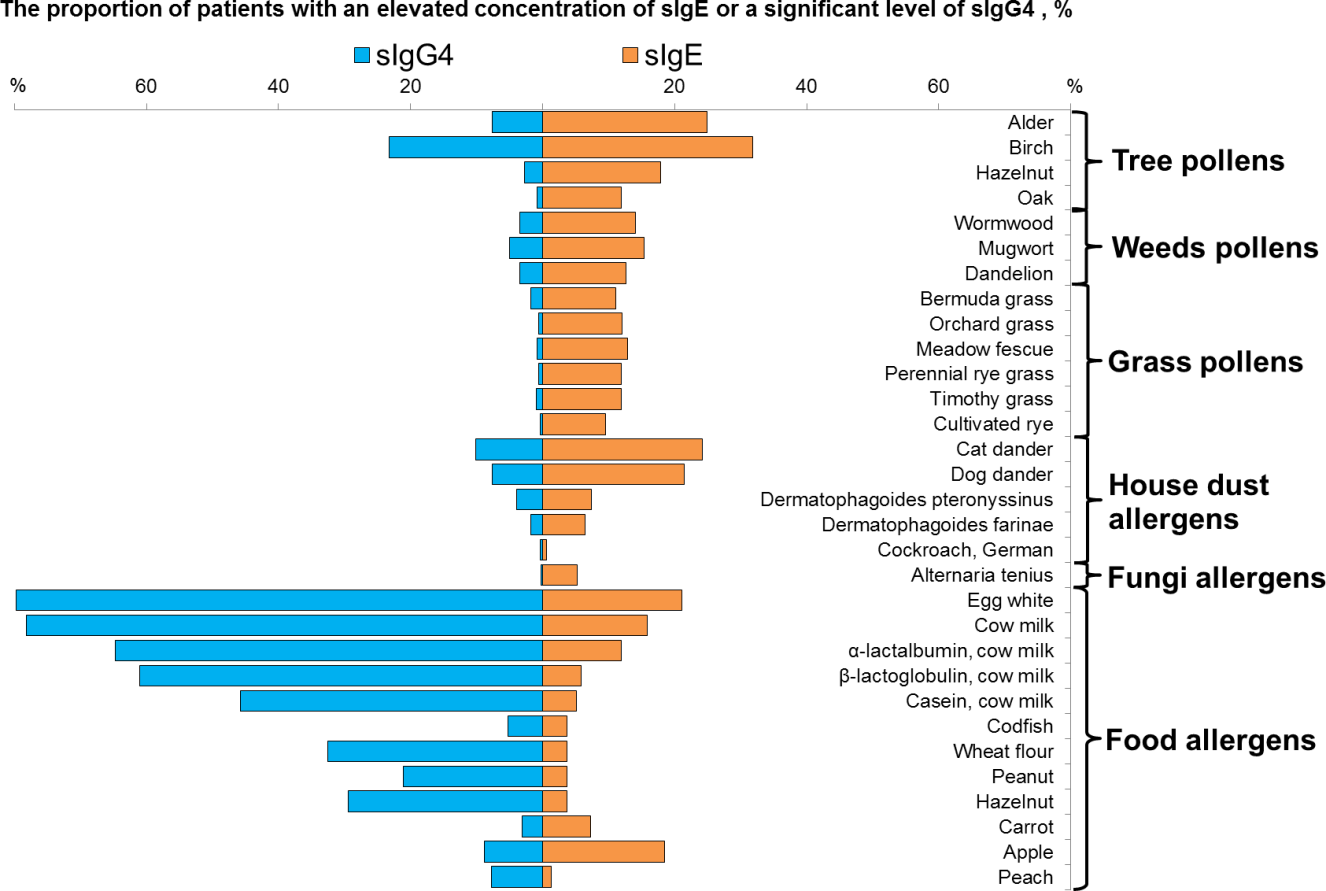
The proportion of pediatric patients with allergic symptoms accompanied by allergen-specific IgE or IgG4 response. Immunoglobulin response was considered to be present in cases of increased concentration of sIgE (≥0.35 IU/ml) or a significant level of sIgG4 (≥110 ng/ml) to allergens.

The number of patients grouped by age in 6 groups with or without increased levels of sIgE and significant levels of sIgG4 is presented in Table 1. Statistically significant male/female differences (p<0.05 according to Fisher exact test) in sIgE and sIgG4 profiles were not detected (data not shown), so the evaluation of immunoglobulin prevalence and age-dependent differences were considered for common groups including both males and females. Detailed information on sIgE and sIgG4 prevalence in relation to each allergen is given in S1 Table and Fig 1.

**Table 1:** The number and percent of samples from patients with or without increased levels of sIgE (cut-off at 0.35 IU/ml) or significant levels of sIgG4 (cut-off at 110 ng/ml) to at least one allergen depending on age and gender (total sample size N = 513).

Groups, ages	Total	Male		Female		Male		Female	
		sIgE	sIgE	sIgE	sIgE	sIgG4	sIgG4	sIgG4	sIgG4
		≥ 0.35 IU/ml	< 0.35 IU/ml	≥ 0.35 IU/ml	< 0.35 IU/ml	≥ 110 ng/ml	< 110 ng/ml	≥ 110 ng/ml	< 110 ng/ml
**0–1**	**81 (16%)**	13 (16%)	26 (32%)	20 (25%)	22 (27%)	26 (32%)	13 (16%)	21 (26%)	21 (26%)
**2–3**	**90 (17%)**	27 (30%)	21 (23%)	28 (31%)	14 (16%)	43 (48%)	5 (6%)	38 (42%)	4 (4%)
**4–5**	**77 (15%)**	27 (35%)	16 (21%)	19 (25%)	15 (19%)	42 (54%)	1 (1%)	32 (42%)	2 (3%)
**6–8**	**85 (17%)**	42 (49%)	16 (19%)	18 (21%)	9 (11%)	56 (66%)	2 (2%)	25 (30%)	2 (2%)
**9–12**	**94 (18%)**	32 (34%)	19 (20%)	30 (32%)	13 (14%)	47 (50%)	4 (4%)	42 (45%)	1 (1%)
**13–17**	**86 (17%)**	39 (46%)	8 (9%)	25 (29%)	14 (16%)	47 (55%)	0 (0%)	39 (45%)	0 (0%)

Fig 1 shows that the most common allergens responsible for sIgE-mediated sensitization were birch pollen (32%), alder pollen (25%), cat dander (24%), dog dander (21%), and egg whites (21%). Other common allergens were grass pollen, cow milk and apples, for which rates of sensitization were in the range of 10-15%.

**Fig 2:**
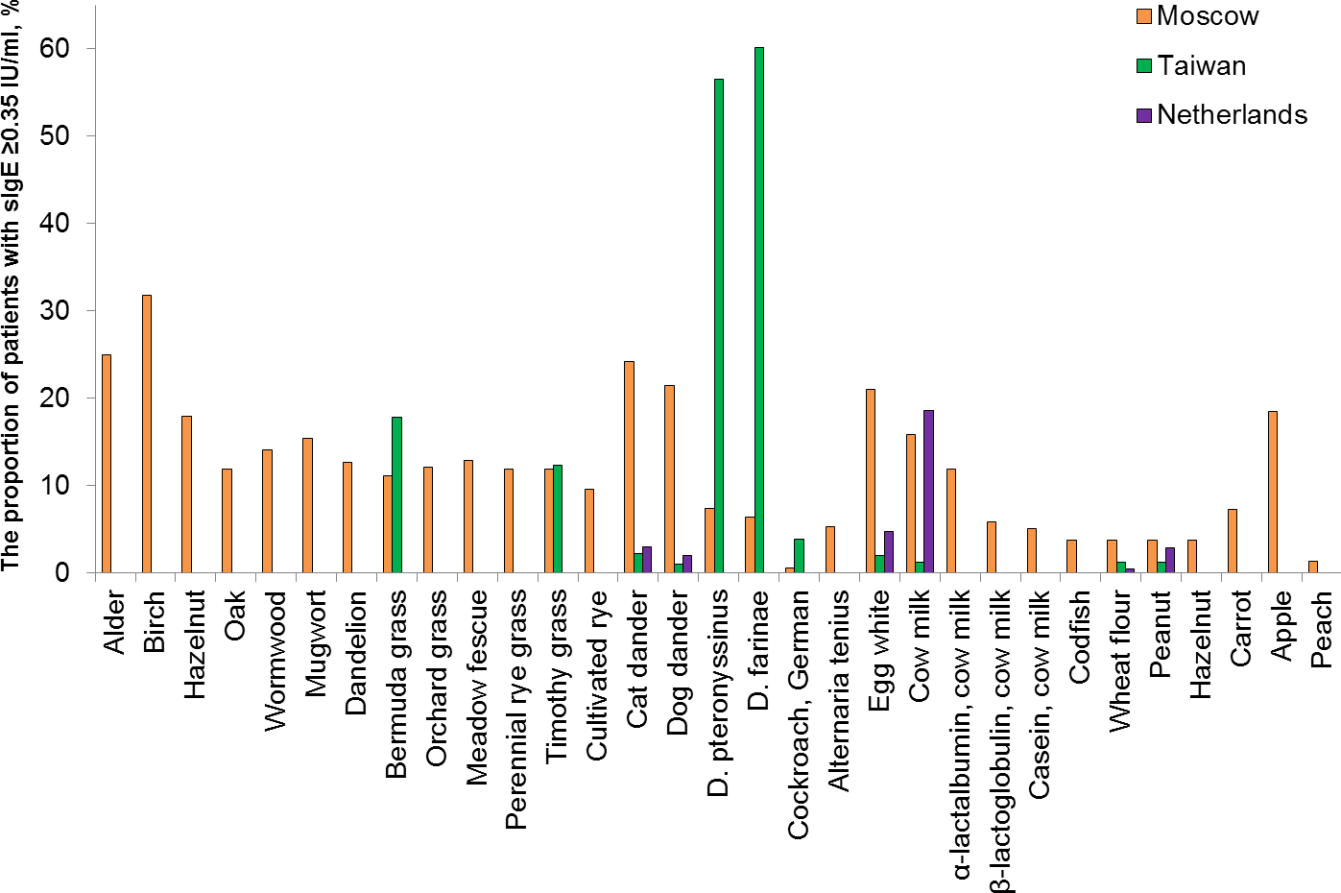
Comparison of sensitization rates from different studies. The proportion of samples with sIgE-increased concentration to each of the 31 allergens for children aged 0 to 17 years according to our data in comparison with the data of other studies conducted in the Netherlands [19] and Taiwan [20].

For comparison, rates of sensitization obtained in the study and the data from the two other investigations that describe sensitization among allergic populations in the Netherlands [19] and Taiwan [20] were added on Fig 2. The diagram shows that the degree of incidence of sIgE to dust mites in the Moscow region (7%) was much lower in comparison with Taiwanese patients (more than 50%). At the same time, rates of sensitization to cat or dog dander and egg whites in the Moscow region were considerably higher than in the other 2 regions of interest. These observations suggest that the external environment has a strong influence on the formation of the set of allergens that cause the majority of the allergic reactions.

As concerns sIgG4 prevalence, sIgG4 antibodies were detected most commonly in response to allergens such as egg whites (80% of patients) and cow milk and its components (46–65%). To a lesser degree, considerable levels of sIgG4 were reached in response to wheat flour (33%), peanuts and hazelnuts (21% and 29%, respectively), and birch pollen (23%) (Fig 1).

**Fig 3:**
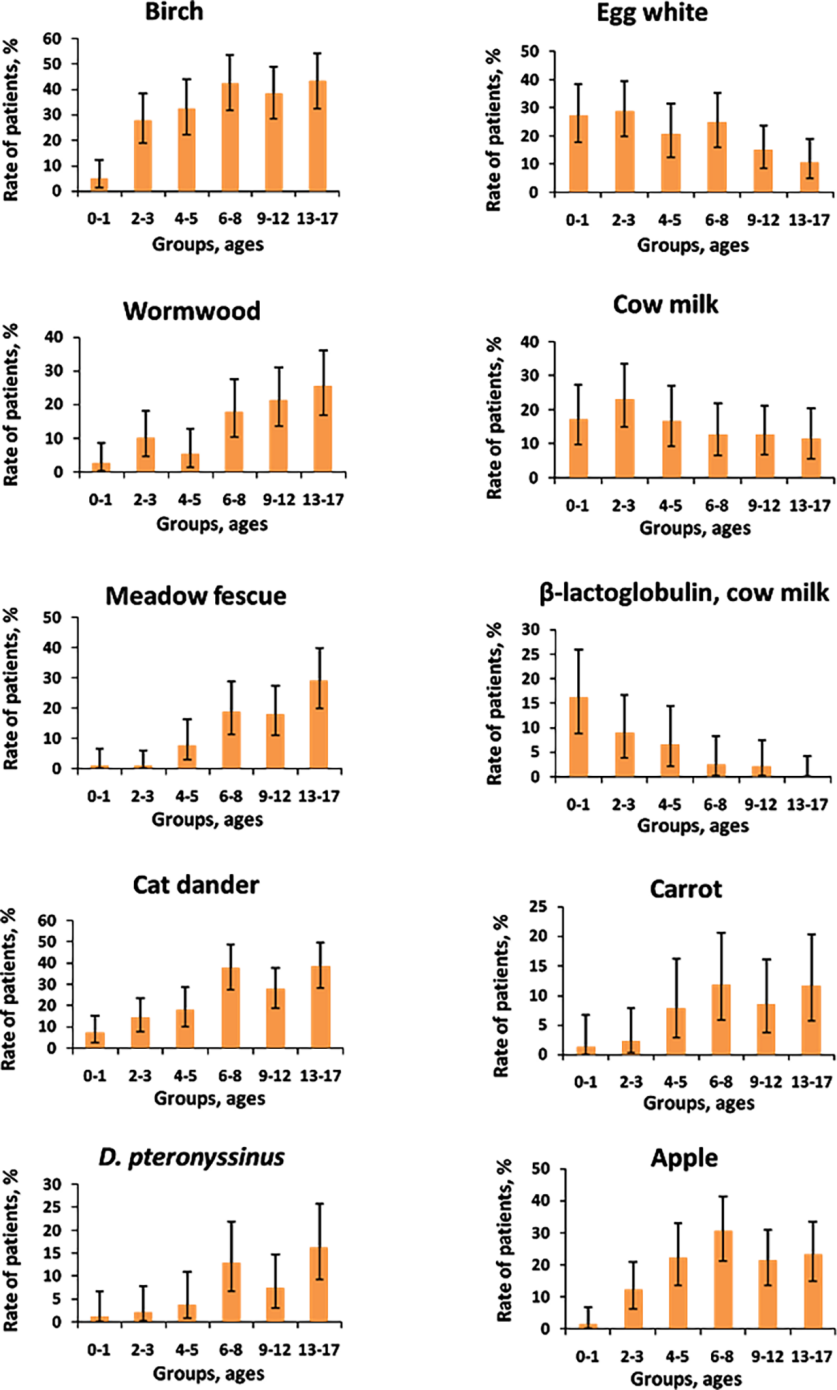
Age-dependent changes in the proportion of patients with increased concentrations of sIgE.

**Fig 4:**
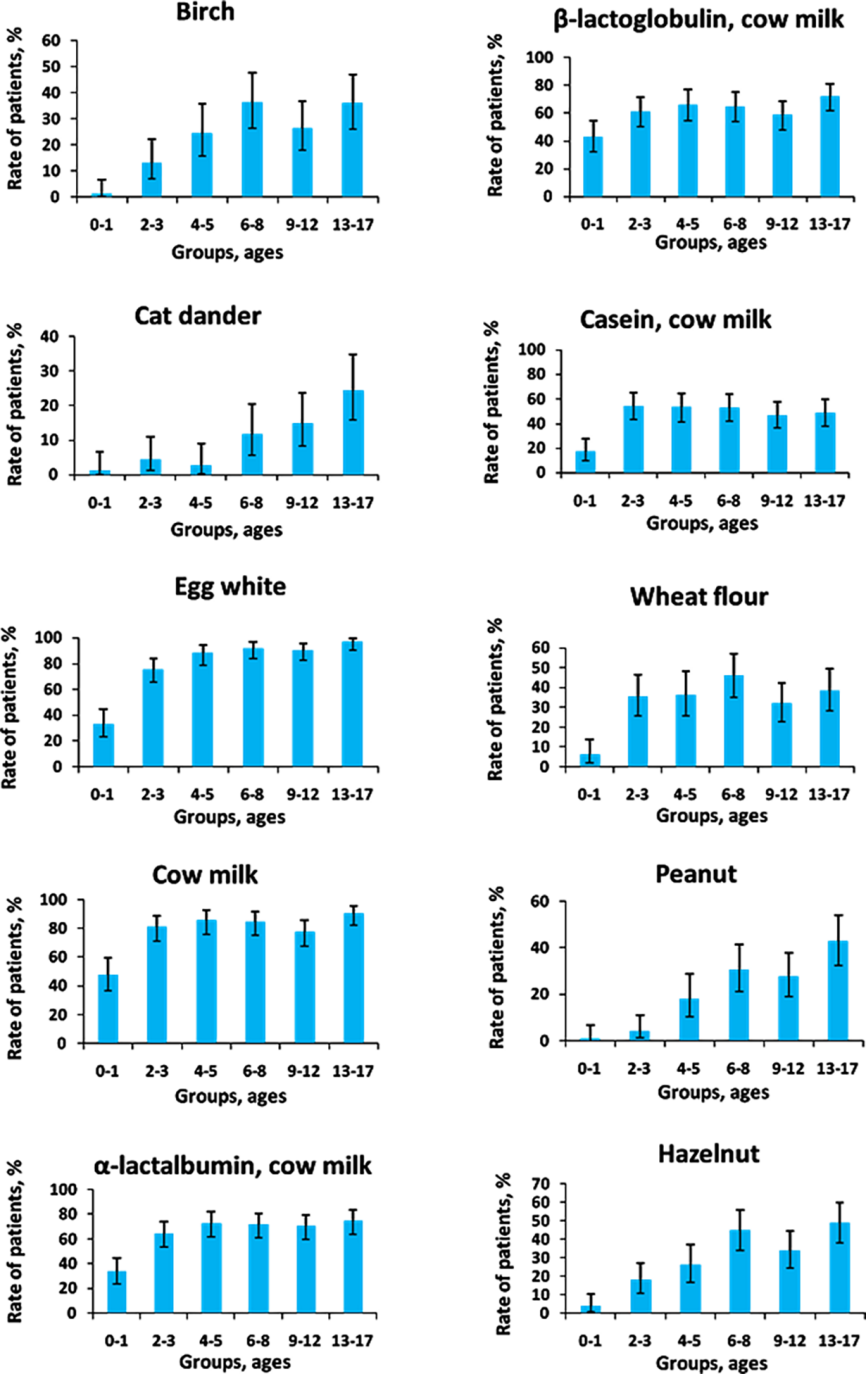
Age-dependent changes in the proportion of patients with significant concentrations of sIgG4.

For most common allergens, the age-dependent changes in sIgE and sIgG4 prevalence were studied. The percentages of patients with significant levels of sIgE and sIgG4 as well as the 95% confidence intervals corresponding to these proportions are shown in Figs 3 and 4. Fig 3 illustrates the age-dependent increase in the ratio of patients with high levels of sIgE to pollen allergens and indoor allergens and the age-dependent decrease for sIgE against most food allergens, except for carrots, apples and peaches. The proportion of patients with significant levels of sIgG4 in response to each allergen increased with the age of the patients (Fig 4).

Pairwise evaluation of statistical significance in sIgE and sIgG4 prevalence between the age groups for observed allergens (S2 Table) has shown significant differences for at least one of groups with younger patients (0–1, 2–3, 4–5) comparing to at least one of the groups with older patients (6–8, 9–12, 13–17) which proves the increasing or decreasing of antibody prevalence with aging.

## Discussion

This study is focused on the analysis of allergen-specific IgE and IgG4 profiles among 513 pediatric patients from the Moscow region who demonstrated allergic symptoms. Most of the previous studies describing allergy occurrence in European countries [21], Asia [22,23], the USA and Canada [24] usually cover only one group of allergens (foods or inhalant allergens) [25,26]. Furthermore, analysis of sensitization profiles for a great number of heterogeneous allergens is carried out in even fewer publications [27].

The major survey on allergies and asthma among children in Russia was carried out as part of The International Study of Asthma and Allergies in Childhood (ISAAC) [28]. The study was mostly focused on revealing and characterizing the allergy symptoms. Additionally, the most critical allergy vulnerability group of children under the age of 6 years was not taken into consideration. Examinations of sensitization profiles among mixed populations of children and adults were performed in Tomsk Oblast [29] and in Saint Petersburg [30].

Inhalant allergens that were included in our study are the most common allergens responsible for allergic rhinitis and rhinoconjunctivitis among Europeans. In Central Europe, sensitization to grass pollen, especially to timothy grass, prevails among pollen allergens [21]. According to our data, sensitization to tree pollens, in particular to birch pollen is prevalent in the Moscow region (Fig 1). This fact could be attributed to the high urbanization in this territory and the significant proportion of these types of trees among the local flora.

Concerning the indoor allergens, cat dander is the prevailing allergen among sensitized children in the Moscow region (Fig 1). Sensitization to cat dander occurred in 24% of the patients in the study. Sensitization to house dust mites (*D*. *pteronyssinus* and *D*. *farinae*) is less common. sIgE response to *D*. *pteronyssinus* was detected only for 7% of the patients included in this study. Also, among the group of older patients aged from 13 to 17 years sensitization to *D*. *pteronyssinus* rises to 16% (Fig 3). It rivals with the percentage of senior schoolchildren showing reaction to dust mites or their main recombinant components in some other European countries, for example, France (9,79% - 26,88% depending on the city [31]) and Poland (18% [32]).

House dust mite allergens are one of the most common allergens in Southeast Asia, Japan, Taiwan and other countries with the rates of sensitization as high as 60%. Such wide difference between dust mite sensitization reported in the studies concerning Asia and a number of European countries could be probably explained both by evaluation of sIgE sensitization in different age groups and by the different food habits including larger shellfish consumption in Asian cuisine. Sensitization to tropomyosin, which is responsible for cross-reactions between shellfish family and house dust mites [33,34], probably results in higher prevalence of sensitization to *D*. *pteronyssinus* and *D*. *farinae* in the regions characterized with increased shellfish consumption [35] (Fig 2).

According to the results obtained in this study, the majority of patients who are sensitized to food allergens are children under the age of 10 years (S1 Table). The most common sensitizing food agents for infants in the Moscow region are egg whites and cow milk (S1 Table), which corroborates the data from other countries across the world [1,36].

Sensitization to peanuts and hazelnuts among atopic children in the Moscow region is rarely observed, compared with allergens such as egg and cow milk. Nevertheless, peanuts and hazelnuts remain very important allergens, as they are responsible for up to 70–90% of all cases of food-induced anaphylactic reactions [37]. The sensitization rates of these allergens vary significantly depending on genetic factors and food habits of the examined patients [38]. For example, the rate of sensitization to peanuts and tree nuts may be up to 12.2% in European countries [39]; while in Asian countries, this type of sensitization is less common, and more significant allergens tend to be soybeans and shellfish [36].

Increased sIgE levels for carrots and apples were observed in 7% and 19% of atopic children that were involved in our study (Fig 1). This significant rate of sensitization can be explained by the high frequency of sensitization to pollen allergens and the cross-reactivity between aeroallergens, especially pollens, and plant food allergens due to the homologous major proteins of the different groups [40].

Age-related changes in the occurrence of sensitization to allergens in various groups of children in the Moscow region (Fig 3) correspond to trends that are observed in populations from other countries of the world [41], where the occurrence of sensitization to inhalant allergens increases with the age of the patients, whereas the number of patients who have sensitization to food allergens decreases with age. One exception, according to the data presented in Fig 3, is apples and carrots, which may show growth in sensitization associated with the birch pollen allergen [42].

In addition to the sIgE profile, sIgG4 profiles were evaluated for all patients. Significant levels of sIgG4 are associated with the formation of tolerance to certain allergens [43] since sIgG4 can act as a blocking antibody, preventing the binding of the allergen to sIgE and further cross-linking of mast cell receptors, followed by the release of inflammatory mediators. Additionally, an increase in the level of sIgG4 is an indicator of a change in Th1/Th2 balance, which in turn leads to a decrease in the synthesis of sIgE and a reduction in clinical symptoms [44].

In our study, the most significant level of sIgG4 was found for food allergens. Apparently, the increased synthesis of sIgG4 is a normal physiological response to consumption of proteins [45]. At the same time, for all of the allergens, there was, without exception, an age-dependent increase in the proportion of patients with a significant level of sIgG4 (Fig 4). This can be explained by the patient's immunological maturation and the ability of the organism to eliminate the specific allergen more quickly and efficiently, thus developing tolerance for food allergens in particular.

In our study, we analyzed the levels of IgE and IgG4 which are specific to the main allergens that cause sensitization in children, as well as the age-dependence of the sensitizing activity of allergens in the Moscow region. Information on the allergens that most often cause sensitization in a particular region may be of importance for effective diagnostics and treatment of atopic patients.

## Supporting information

S1 Table Number of patients with sIgE and sIgG4 response and prevalence (%) of specific antibodies in different age groups. Conditional color formatting was used for the lines with percentages (from green color for the lowest value to red color for the highest value).
(XLSX)

S2 Table P-values obtained by Fisher’s exact test for pair-wise comparisons of antibody prevalence in different age groups. (XLSX)
